# Exploring the causal association between rheumatoid arthritis and the risk of cervical cancer: a two-sample Mendelian randomization study

**DOI:** 10.1186/s13075-023-03240-2

**Published:** 2024-01-23

**Authors:** Minxian Xu, Huan Chen, Tao Tan, Kaihong Xie, Hui Xie, Qing Li

**Affiliations:** 1 Present Address: Department of Oncology, Affiliated Hospital (Clinical College) of Xiangnan University, Chenzhou, 423000 People’s Republic of China; 2 Department of Gynecology, Affiliated Hospital (Clinical College) of Xiangnan University, Chenzhou, 423000 People’s Republic of China; 3 Faulty of Applied Sciences, Macao Polytechnic University, Macao, 999078 People’s Republic of China; 4 Department of Radiation Oncology, Affiliated Hospital (Clinical College) of Xiangnan University, Chenzhou, 423000 Hunan Province People’s Republic of China; 5 School of Medical Imaging, Laboratory Science and Rehabilitation, Xiangnan University, 423000 Chenzhou, Hunan Province People’s Republic of China

**Keywords:** Causal relationship, Rheumatoid arthritis, Cervical cancer, Mendelian randomization

## Abstract

**Objective:**

Whether rheumatoid arthritis patients have an increased risk of cervical cancer remains controversial, and further research is needed on this clinical question. This study aims to investigate the association between rheumatoid arthritis and the susceptibility to cervical cancer by employing Mendelian randomization methodology, utilizing the extensive dataset from human genome-wide association data analysis.

**Methods:**

The publicly accessible MR base database was utilized to obtain the complete genome, relevant research findings, and summarized data pertaining to rheumatoid arthritis and cervical cancer. Genetic tool variables, specifically single-nucleotide polymorphisms closely linked to rheumatoid arthritis, were chosen for analysis. Four methods, namely inverse variance weighted analysis, weighted median analysis, weighted mode, and MR-Egger regression, were employed. Statistical analysis was conducted to explore the potential association between rheumatoid arthritis and susceptibility to cervical cancer.

**Results:**

The results of the inverse variance weighted analysis (OR = 1.096, 95% CI: 1.018–1.180, *P* = 0.015) indicate a significant causal relationship between rheumatoid arthritis and an increased risk of cervical cancer. Furthermore, the absence of horizontal pleiotropic effects (MR-Egger intercept = 0.00025, *P* = 0.574) and heterogeneity (QEgger = 2.239, I2Egger = 0.225, PEgger = 0.268, QIVW = 2.734, I2IVW = 0.220, PIVW = 0.999) suggests that the observed association is not influenced by confounding factors. Sensitivity analysis and other statistical methods also support the conclusion that genetic pleiotropy does not introduce bias to the findings.

**Conclusion:**

There is a causal relationship between rheumatoid arthritis and the occurrence of cervical cancer. People with rheumatoid arthritis is one of the high-risk groups for early screening of cervical cancer. The IL-18 may play a significant role in elevating the risk of cervical cancer among rheumatoid arthritis patients.

## Background

Cervical cancer (CC) is a prevalent malignancy globally, ranking third in terms of occurrence and fourth in terms of mortality among women [1]. According to global cancer data published by the International Agency for Research on Cancer (IARC) in 2020, there were 604,127 new cases of CC and 341,831 new deaths worldwide [1]. The primary etiology of CC is attributed to persistent human papilloma virus (HPV) infection, which is detected in nearly 99% of CC cases, with the high-risk subtypes HPV16 and 18 being the most prevalent [2].

In recent years, there has been a decrease in the global prevalence of CC due to the implementation of CC screening and vaccination programs. However, it is important to note that the incidence of CC still surpasses 85% [3] and accounts for nearly 90% of deaths in developing nations [3]. A recent report indicates that China recorded 119,000 newly diagnosed cases of CC in 2020, and the incidence rate continues to rise [4]. Consequently, CC remains a significant health concern that poses a threat to women’s well-being in China.

A population of patients with rheumatoid arthritis (RA) has been found to have a notable susceptibility to HPV infection and cervical dysplasia, as reported in previous studies [5]. Extensive cohort studies conducted in Western Europe and North America have demonstrated that female RA patients face a 1.3–1.5 times higher risk of developing significant cervical ectasia compared to women without RA [6]. Nevertheless, the findings of a meta-analysis yielded contrasting results, indicating that RA patients did not exhibit an elevated risk of developing CC [5]. Therefore, whether RA patients have an increased risk of CC remains controversial, and further research is needed on this clinical question.

The presence of reverse causality and measurement error in previous observational studies investigating the relationship between RA and CC has hindered the establishment of consistent conclusions. While well-designed randomized controlled trials (RCTs) are regarded as the optimal approach in clinical research to mitigate the limitations of observational studies, they too possess certain constraints. Therefore, in order to enhance the level of evidence-based medicine and ascertain the precise role of RA in CC pathogenesis, further investigation is warranted. A more precise methodology is required for the execution of scientific studies. Mendelian randomization (MR) analysis [7] is an analytical approach that examines causal connections between genotypes at the genetic level. MR analysis employs genetic variation as an instrumental variable (IV) for the exposure factor in order to evaluate whether alterations in the exposure variable have a direct impact on the outcome, thereby investigating the causal association between the exposure factor and the outcome. Moreover, as a result of the Mendelian random assignment that takes place during the allocation of single-nucleotide polymorphisms (SNPs) to offspring at conception, which consistently happens prior to the development of disease, the susceptibility of analysis using MR to be influenced by exposure is diminished. Consequently, MR analysis is less prone to the constraints encountered in prior observational studies. In this particular investigation, we employed MR analysis to examine the genetic causality between RA and CC, ascertain the risk factors associated with CC development, and furnish fresh evidence regarding the involvement of RA in the progression of CC. We hope to provide clinical evidence for the elucidation of the mechanism of CC development and the prevention and treatment of CC.

## Material and methods

### Research design

**Fig. 1:**
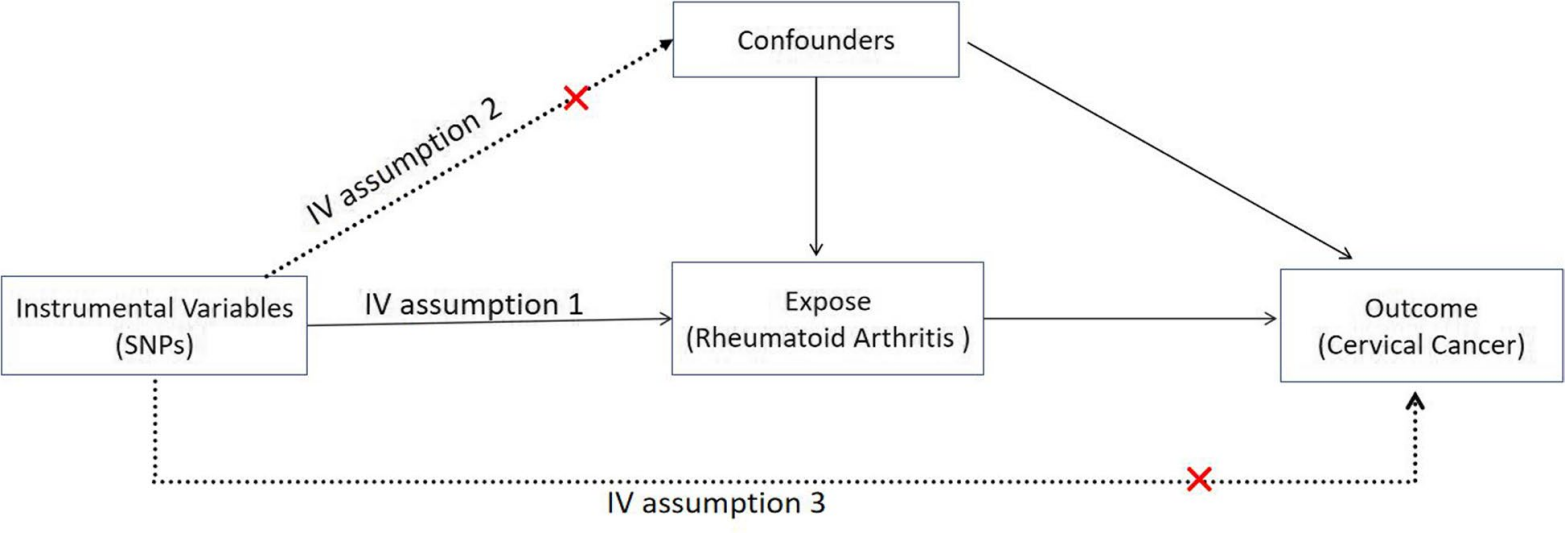
Three assumptions for IVs in MR analysis

In this study, we evaluated RA as an exposure variable and examined the occurrence or absence of CC as an outcome event. To conduct our analysis, we utilized a genome-wide association study (GWAS) genetic dataset for two-sample MR analysis. We aimed to investigate the potential causal relationship between RA and CC. The schematic representation of the MR study design can be observed in Fig. 1.

The following assumptions need to be met in order to perform MR analysis: (A) the identified SNPs must be highly correlated with the exposure factor, (B) the SNPs should be independent of any confounding variables, and (C) the SNPs can only influence the outcome through exposure.

### Source of data

The RA dataset was acquired from the publicly available database MR base (https://app.mrbase.org/) [8]. The data was published by the Neale lab et al. in 2018 with the ID ukb-d-M06. The dataset included both male and female subjects, consisting of 361,194 enrolled participants, with 1401 patient cases, 359,793 controls, and 9,944,222 SNPs variables. Significantly, all the enrolled participants were of European descent.

The CC dataset, identified with the ID ieu-b-4876, is sourced from the public database MR base (https://app.mrbase.org/) [8]. Burrows et al. released this dataset in 2021. It specifically focuses on CC and consists of data from 199,086 subjects. Among these subjects, 563 were CC patients, while 198,523 served as controls. The dataset includes information on 850,621 SNPs variables. Similar to the RA dataset, the CC dataset enrolls individuals exclusively from European populations.

### SNP screening

In the analysis of MR, SNPs present in the exposure dataset were employed as IVs. For this particular study, the SNPs that were selected from the acquired and GWAS data had to exhibit a high correlation with the exposure (*P* < 5 × 10^−8^). Additionally, to ensure that the IVs satisfied the assumptions of the MR analysis, a linkage disequilibrium parameter of *R*^2^ < 0.01 was set at a genetic distance of 10,000 Kb. Furthermore, the strength of each SNP was evaluated using *F*-statistic values, and any SNPs with *F*-statistic values below 10 were excluded [9]. Only SNPs that were present in both the exposure and outcome GWAS datasets were investigated in this MR study, excluding proxy SNPs [10]. The *F*-statistic values and R were calculated as in formulas 1 and 2. Finding candidate gene SNPs using NCBI-SNP (http://www.ncbi.nlm.nih.gov/snp/).

1 $$F-Static=\frac{\left[{R}^{2}\left(N-2\right)\right]}{\left(1-{R}^{2}\right)}$$

2 $${R}^{2}=\frac{2*{\left(Beta\right)}^{2}*EAF*\left(1-EAF\right)}{2*{\left(Beta\right)}^{2}*EAF*\left(1-EAF\right)+2*{\left(SE\right)}^{2}*N*EAF*\left(1-EAF\right)}$$

N: the number of samples exposed to GWAS studies; *R*^2^, the degree to which IV explains exposure (the determinant of the regression equation); Beta, the effect size of each allele for each SNP and phenotypic association; SE, the standard deviation of Beta, EAF, the effect allele frequency.

### Statistical analysis

This study employed four statistical methods, namely inverse variance weighted (IVW) analysis [11], weighted median [12], MR-Egger regression [12], and weighted mode [13], to examine the causal relationship between RA and the risk of CC. The IVW analysis method, being the most traditional MR research approach, assigns appropriate weights to the 4 SNPs when all SNPs meet the criteria of valid IVs. The application of the weighted median method necessitates that a minimum of 50% of the SNPs satisfy the necessary conditions for a valid IV. Subsequently, the included SNPs are arranged in ascending order based on their respective weights, and the resulting analysis yields the median value of the associated distribution function. Weighted mode analysis, on the other hand, serves as a data sampling technique employed to rectify the imbalanced distribution of samples across various categories in the context of disparate datasets. The MR-Egger regression analysis successfully estimated the causal effect of the outcome, even in the presence of genetic pleiotropy in the included SNPs. The slope of the regression represents the estimated causal effect of RA on the risk of CC. However, due to its limited test efficacy and wide confidence intervals, the MR-Egger analysis is commonly employed as a sensitivity analysis for other statistical findings. All results were expressed as odds ratio (OR) and its 95% confidence interval (CI), and *P* < 0.05 was considered statistically significant. Statistical analysis was done in R software 3.4.2.

### Sensitivity analysis

#### Heterogeneity test

The heterogeneity test is employed to assess the presence of variation among independent IVs. The magnitude of this variation directly corresponds to the level of heterogeneity among the IVs. The Cochran’s *Q* statistic [14] is used to measure heterogeneity by calculating a weighted sum of squared distances between specific estimates of the IV and the overall IVW estimate.

#### Pleiotropic test

MR-Egger’s intercept can be used to test whether the IVs are horizontal pleiotropic [15]. MR-Egger assumes the presence of an intercept term in the model; if the intercept term is close to zero, the estimated causal effects from both MR-Egger and IVW are closely similar, indicating the absence of horizontal multicollinearity among the IVs. If the intercept term is very different from zero, then horizontal pleiotropic among the IVs can be indicated.

#### Leave-one-out test

The leave-one-out test is a technique wherein SNPs are systematically eliminated from the analysis, allowing for the re-estimation of the causal effect associated with the removal of each individual SNP [16]. If, after removing an SNP, the remaining IVs significantly deviate from the non-eliminated IVs, it suggests that this SNP has a significant influence on the causal estimation, thus rendering the results non-robust.

## Results

### Screening of instrumental variables

After screening for RA statistics, four SNPs were entered into the study, and the specific data are shown in Table 1. The sum of the *R*^2^ of all IVs was 0.0031, explaining 0.31% of the risk of CC, and the values of the *F*-statistics ranged from 65 to 632, all of which were greater than 10, suggesting that the likelihood of the existence of a weak instrumental bias was low. These SNP IDs were retrieved from the NCBI’s SNP database (http://ncbi.nlm.nih.gov/snp/). The gene corresponding to rs35139284 and rs35511257 is major histocompatibility complex, class II, DR beta 1 (HLA-DRB1). The gene corresponding to rs41270903 is major histocompatibility complex, class II, DQ beta 1 (HLA-DQB1), and the gene corresponding to rs6679677 is putative homeodomain transcription factor 1 (PHTF1).

**Table 1:** Detailed information on SNPs associated with rheumatoid arthritis and cervical cancer

Rsid	Chr	EA	OA	Pos		Exposure					Outcome					
					EAF	*β*	SE	*P*	*R*^2^	*F*	EAF	*β*	SE	*P*	*R*^2^	*F*
rs35139284	6	T	C	32561370	0.320	0.004	1.615E − 04	1.72E − 106	1.70E − 03	613.319	0.317	1.759E − 04	1.814E − 04	0.330	4.72E − 06	0.940
rs35511257	6	C	G	32545392	0.095	0.005	2.742E − 04	1.29E − 60	9.20E − 04	332.576	0.092	5.747E − 04	3.115E − 04	0.065	1.71E − 05	3.404
rs41270903	6	G	A	32629618	0.193	0.002	2.037E − 04	3.71E − 21	2.67E − 04	96.419	0.180	1.781E − 04	2.190E − 04	0.420	3.32E − 06	0.662
rs6679677	1	A	C	114303808	0.102	0.002	2.475E − 04	1.51E − 12	1.81E − 04	65.290	0.101	5.343E − 04	2.793E − 04	0.056	1.84E − 05	3.660

*Rsid* identification code of reference single nucleotide polymorphism, *Chr* chromosome, *Pos* position, *EA* effect allele, *OA* other alleles, *EAF* effect allele frequency, *SE* standard error

### Results of Mendelian randomization

Table 2 shows the results of the four MR methods for estimating the causal effect between RA and CC. The results of IVW suggested that having RA increased the risk of CC (OR = 1.096, 95% CI: 1.018–1.180, *P* = 0.015). The results of weighted median indicated the same conclusion (OR = 1.083, 95% CI: 0.999/1.174, *P* = 0.054). Although the MR-Egger method (OR = 1.015, 95% CI: 0.800/1.288, *P* = 0.911) and weighted mode (OR = 1.066, 95% CI: 0.978/1.163, *P* = 0.240) did not show statistical significance due to low statistical power, it showed the same direction of the effect as the other methods and also shows a trend that having RA increases the risk of developing CC.

**Table 2:** Mendelian randomization results in rheumatoid arthritis and cervical cancer

Method	Nsnp	*β*	SE	OR	Low 95%	Up 95%	*P*
MR Egger	4	0.015	0.122	1.015	0.800	1.288	0.911
Weighted median	4	0.070	0.041	1.083	0.999	1.174	0.054
IVW	4	0.092	0.038	1.096	1.018	1.180	0.015
Weighted mode	4	0.064	0.044	1.066	0.978	1.163	0.240

*Nsnp* the number of SNP, *SE* standard error, *OR* odds ratios, *Beta* the effect size of each allele for each SNP and phenotypic association

**Fig. 2:**
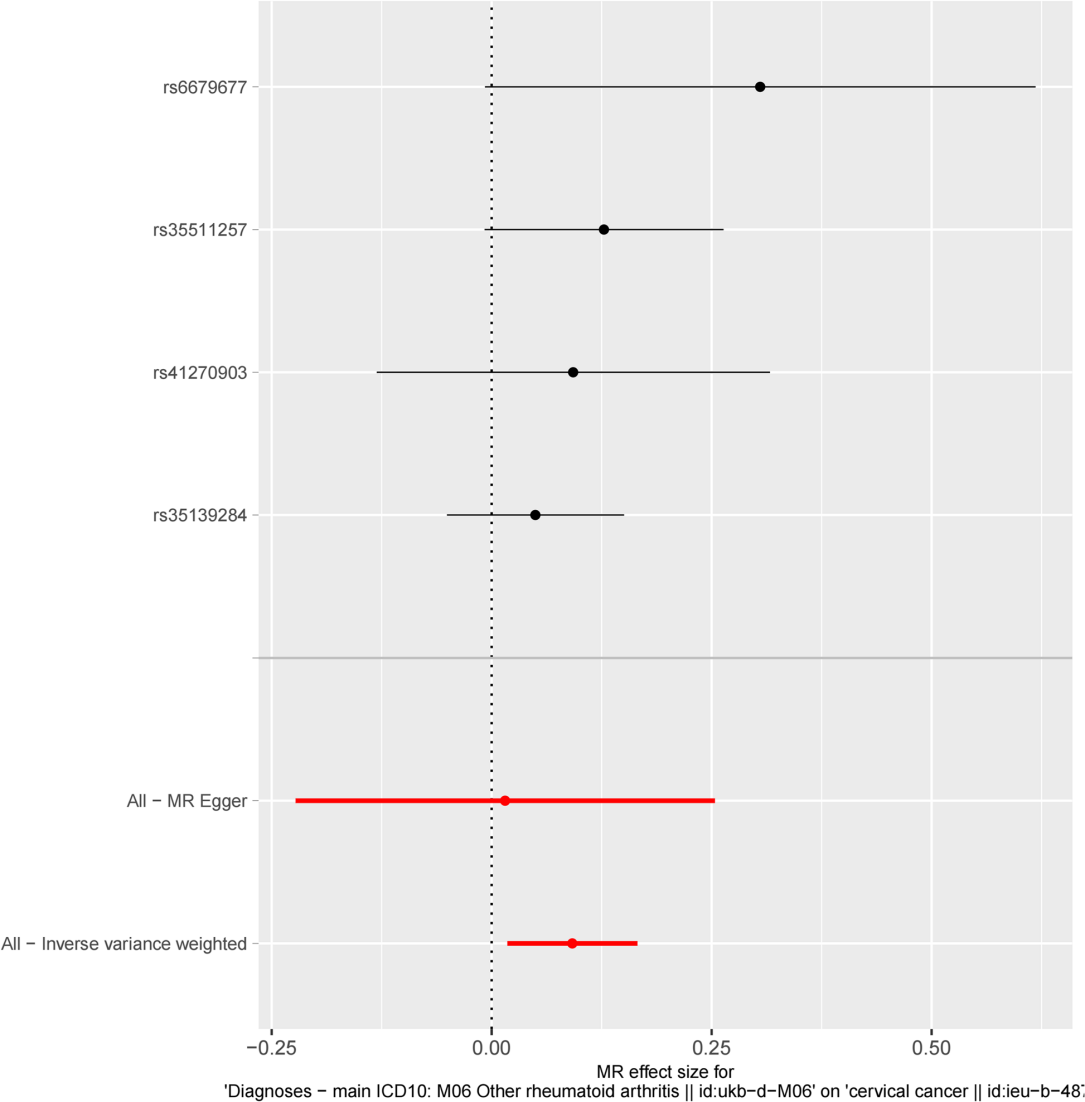
Forest plot of estimated causal effects of rheumatoid arthritis -related SNPs on cervical cancer

Simultaneously, we conducted calculations and generated visual representations of the causal effect estimates pertaining to the individual IVs as well as the overall summary. The outcomes of the four statistical methods employed in MR analysis, along with the causal effects associated with the individual IVs, are succinctly summarized in Fig. 2, which was presented in the form of a forest plot. The dots within the plot signify the calculated *β* values, while the horizontal line represents the 95% CI of *β*. The red line signifies the estimated causal effect derived from the MR methods. The figure demonstrated that the horizontal line corresponding to the IVW method didn’t not intersect the dashed line, thereby indicating its statistical significance.

**Fig. 3:**
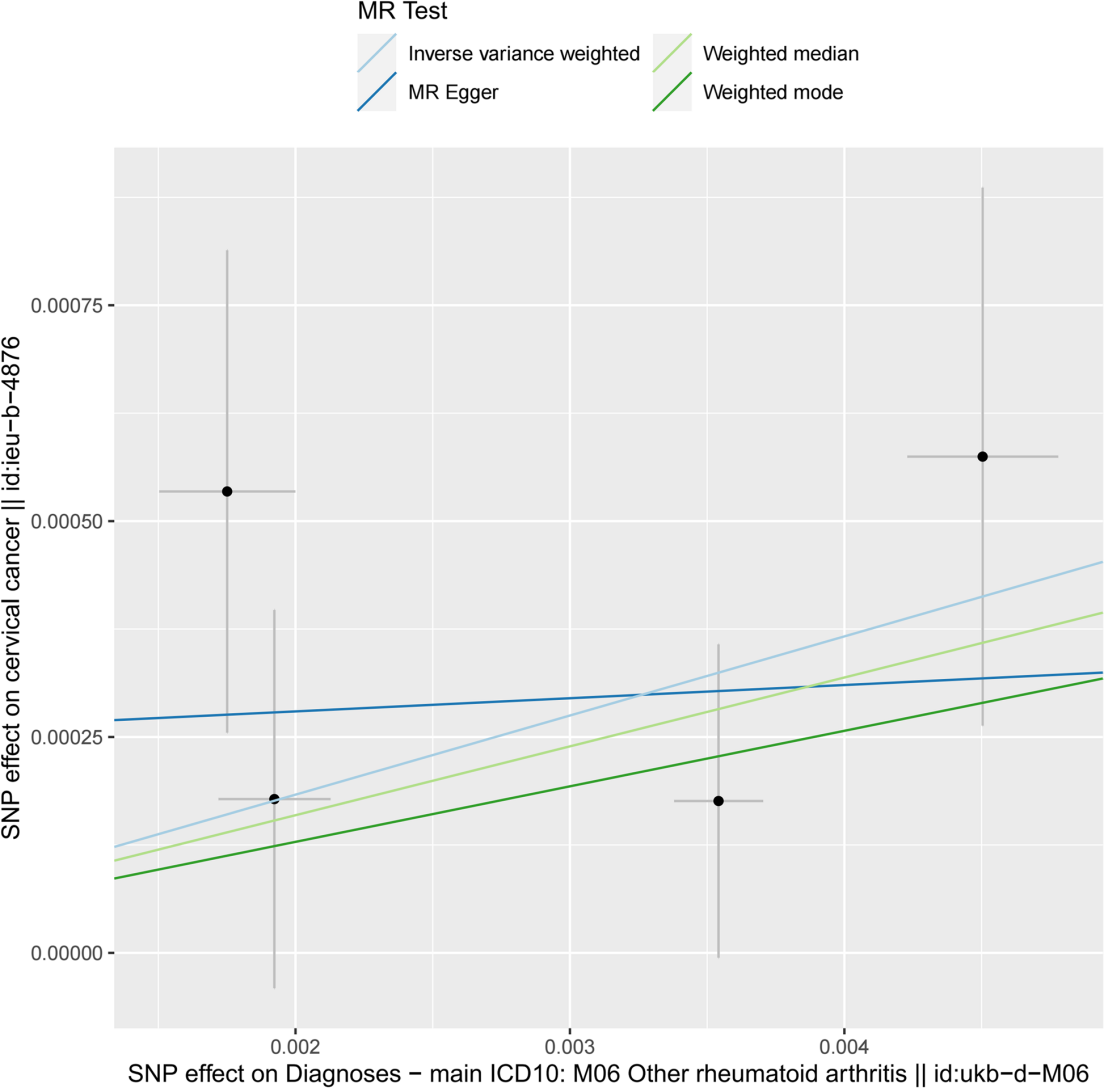
Scatterplot of estimated causal effects of rheumatoid arthritis-related SNPs on cervical cancer

Figure 3 displayed scatter plots illustrating the causal effect estimates for the SNP in isolation as well as the four MR methods. Each IV was represented by a black dot, with the horizontal coordinates denoting the effect of the SNP on RA and the vertical coordinates indicating the effect of the SNP on RA. The extended vertical and horizontal lines correspond to the 95% CI of the causal effect of the IV on the respective disease. The slopes of the four colored diagonal lines represent the estimated values of the causal effects obtained through the four MR methods. The results depicted in Fig. 3 demonstrated that the slopes of the four MR methods exhibit positive values, indicating that an increase in the effect of RA corresponds to an increase in the effect of CC. While the statistical significance was not achieved for the MR-Egger, weighted median, and weighted mode methods, they nonetheless exhibited a consistent pattern suggesting that RA may elevate the risk of CC.

#### Sensitivity analysis

The results of Cochran’s *Q* test implied that there was no heterogeneity in the effect estimates for all IVs (Q_Egger_ = 2.239, I^2^_Egger_ = 0.225, P_Egger_ = 0.268, Q_IVW_ = 2.734, I^2^_IVW_ = 0.220, P_IVW_ = 0.999, Table 3).

**Table 3:** Cochran’s *Q* test results

Method	*Q*	df	*I*^2^	*P*
Inverse variance weighted	2.734	2	0.220	0.999
MR Egger	2,239	2	0.225	0.268

**Fig. 4:**
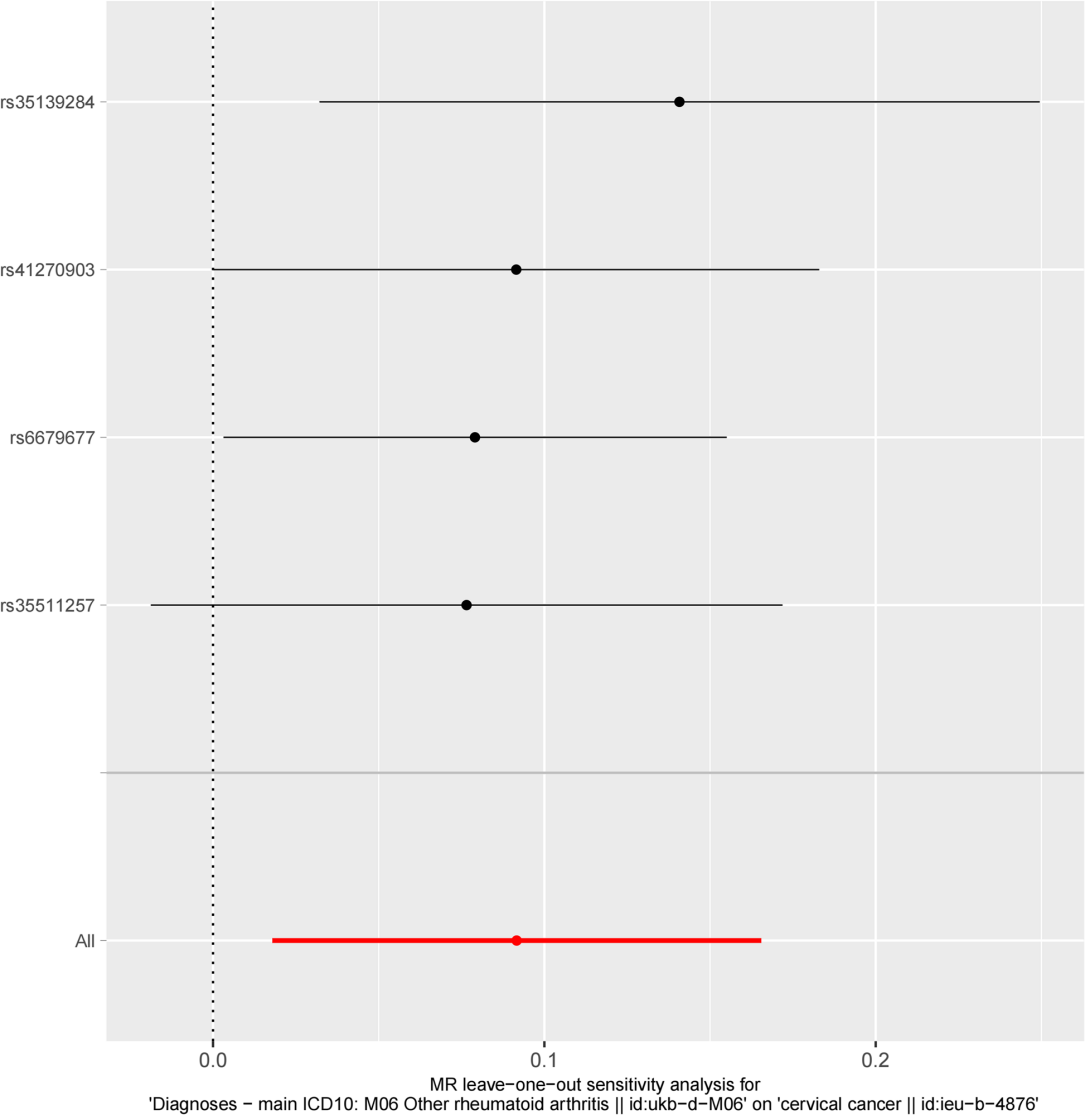
Mendelian randomization funnel plot

The MR-Egger regression intercept yielded a non-significant result (MR-Egger intercept = 0.00025, *P* = 0.574), indicating the absence of directional pleiotropy in the relationship between RA and CC. Additionally, the leave-one-out test (Fig. 4) did not demonstrate a statistically significant effect. The forest plot displays the impact of all remaining SNPs (represented by the black line) after excluding the corresponding SNP, while the red line represents the combined effect without removing any SNP. The *β* coefficients and 95% CI are represented by dots and horizontal lines, respectively. Figure 4 demonstrated that the method eliminates any SNP to conduct an IVW effect analysis solely on the remaining SNPs serving as genetic instruments. This approach aims to ascertain the independent influence of the SNPs on the analysis outcomes. The analysis results indicate that all OR values exceed 1, suggesting that individual SNPs did not exert a substantial impact on the estimation of causality. Consequently, the findings were deemed robust and unaffected by the presence of a single SNP.

## Discussion

This study aimed to investigate the causal relationship between RA and the susceptibility to CC using a two-sample MR approach. The findings of this study revealed that RA was significantly associated with an increased risk of CC [OR = 1.096, 95% CI: 1.018–1.180, *P* = 0.015). These MR results underscore the significance of improving screening and preventive measures for CC in individuals diagnosed with RA, in order to promptly identify both CC and precancerous lesions.

This study is similar to many previous studies showing a 1.5 times higher risk of high-grade cervical atypia and CC in women with RA [6]. A study by Rojo et al. concluded that autoimmune diseases, such as RA, are a risk factor for CC [17]. According to Wadstrom et al., CC incidence is higher in women with RA than in the general population over a 12-year follow-up period [18]. Several studies have indicated a notable increase in antibodies against citrullinated HPV among patients with RA, implying a potential association between HPV and the onset of RA [19]. Additionally, it has been established that HPV plays a definitive role in the development of CC [20]. Nevertheless, it is important to note that the majority of existing clinical studies were retrospective in design, rendering them vulnerable to reverse causality and the influence of confounding variables. In this study, MR was used to explore the study of RA and the risk of CC. The findings suggest that patients with RA have a higher risk of developing CC.

According to the findings of the present study, it was observed that a majority of patients diagnosed with RA utilize systemic immunosuppressive or steroid medications [21]. Furthermore, research has indicated a potential correlation between the utilization of systemic immunosuppressants or steroids and the heightened risk of developing CC. Specifically, the study demonstrates that women with RA who are prescribed immunosuppressants exhibit an increased susceptibility to CC when compared to the general population [18].

The study identified four SNPs through MR analysis, which corresponded to three genes: HLA-DRB1, HLA-DQB1, and PHTF1. Notably, two of these genes are located in the HLA II region. HLA class II molecules, characterized by CD4 + , play a significant role in both innate and adaptive immune responses and exhibit a complex relationship with cancer risk [22]. Therefore, it can be inferred that RA-induced CC is likely to be associated with HLA class II molecules. Interleukin-18 (IL-18), produced by various immune and non-immune cells, is known to be involved in the abnormal activation of CD4 + T cells [23], which is associated with the increased expression of IL-18 in the body. More specifically, IL-18 serves as an IFN-γ inducing factor, directly causing CD4 + T cells and CD8 + T cells to highly express IFN-γ [24]. The pathogenesis of RA has been linked to the overexpression of IL-18 and the relative deficiency of IL-18-binding protein levels in RA patients [25]. Since its initial identification, IL-18 has been consistently linked to human HPV infection and CC [26]. Extensive research has demonstrated that variations in the IL-18 gene can significantly impact its expression and functionality, consequently influencing the susceptibility to CC [27]. Specifically, specific genetic variants have been found to result in diminished IL-18 expression [28], thereby elevating the likelihood of developing CC. Conversely, elevated levels of IL-18 have been linked to the eradication of HPV infection and bolstered immune response, consequently diminishing the susceptibility to CC [26]. Furthermore, IL-18 has been observed to exhibit significant expression in CC cells, with its level of expression being positively correlated with the malignancy extent of CC [29]. Several investigations have additionally demonstrated that the modulation of IL-18 expression can exert an influence on the proliferation and apoptosis of CC cells [29]. Furthermore, research has demonstrated that the inhibition of IL-18 binding to its receptor a-chain effectively hinders IL-18-induced interferon-gamma (IFN-γ) production by monocytes and natural killer (NK) cells [30]. IFN-γ, a key component in the immune response against intracellular infections [31], is thus implicated in the heightened susceptibility to CC in patients with rheumatoid arthritis, which can be attributed to prolonged usage of immunosuppressive medications and dysregulation of cytokines and chemokines.

This study aimed to investigate the causal relationship between RA and the risk of CC through the application of two-sample MR analysis, which offers several advantages. Firstly, the inclusion of a larger sample size in this study enhances the credibility of the obtained results. Secondly, the utilization of multiple statistical methods, which yield consistent outcomes, ensures the robustness of this study and strengthens the level of certainty in establishing a causal inference. Lastly, in comparison to conventional retrospective studies, this research effectively mitigates the influence of reverse causality and confounding factors. However, there are still some limitations in this study; the databases used in this study were all from the European region, and their global applicability has yet to be demonstrated. Therefore, there is a need to further investigate the relationship between RA and cervical carcinogenesis in other ethnic populations.

## Conclusion

In summary, this study conducted a comprehensive examination of the potential causal relationship between RA and CC through the implementation of a two-sample MR study. The findings revealed a positive correlation between RA and the susceptibility to CC, suggesting that targeted screening of female RA patients could potentially serve as a preventive measure against CC. Additionally, it was observed that IL-18 may play a significant role in elevating the risk of CC among RA patients. However, further ex vivo experiments are necessary to fully comprehend the underlying biological mechanisms, with the ultimate goal of reducing the occurrence and prevalence of CC.

## Data Availability

All data generated or analyzed during this study are included in this public database: https://app.mrbase.org/ (ID are ukb-d-M06 and ieu-b-4876).

## Abbreviations

MR: Mendelian randomization
GWAS: Genome-wide association data analysis
SNP: Single-nucleotide polymorphisms
IVW: Inverse variance weighted analysis
RA: Rheumatoid arthritis
CC: Cervical cancer
IARC: International Agency for Research on Cancer
HPV: Human papilloma virus
RCT: Randomized controlled trials
IV: Instrumental variable
CI: Confidence interval
OR: Odds ratio
IL-18: Interleukin-18
NK cell: Natural killer cell
IFN-γ: Interferon-gamma
